# Buttermilk as an Infant Food

**Published:** 1907-05

**Authors:** 


### BUTTERMILK AS AN INFANT FOOD.

---

Strauch believes that pathogenic bacteria (of diphtheria, typhoid, tuberculosis, Bacillus pyocyaneus), as a rule, gradually perish spontaneously in buttermilk, both on account of the increasing acidity and the presence of the Bacillus acidi lactici, or they are easily destroyed by boiling for only one, two, to three minutes. We further know that the acidity of the food promotes tryptic digestion, as the acidity of the stomach contents energetically stimulates the secretion of trypsin. The digestibility of the nitrogenous substances of buttermilk lessens the work of the glands of the stomach and the intestines; the amount of energy thus spared is beneficial to the organism of the infant. As to the indications for the use of buttermilk, no absolute rules can be formed, nor is any strict classification of the various intestinal disorders unanimously accepted. Buttermilk has been used with best results in insufficiency of fat—and of albumin—digestion; in atrophia infantum, dependent on chronic enteric catarrh; in dystrophia infantum without determinable causes; in allaitement mixte, and in cases of sudden weaning. The high calorimetric value of the buttermilk renders it fit for feeding prematurely born babies who are not able to nurse, or if no wet nurse can be promptly secured. Many babies with intestinal diseases digest only buttermilk; others digest other foods as well. As a rule, acute intestinal disorders, and those chronic disturbances due to prolonged starch feeding, should be excluded. In case of intolerance for carbohydrates, buttermilk without flour, or even without sugar, may be tried for a short time. The objections against a buttermilk regime are due to the fact that buttermilk of standard quality, which is indispensable for success, can be obtained only with great difficulty in large cities. The common commercial so called buttermilk is indeed too often unfit for infant feeding on account of its contaminations, its unhygienic manipulation, and its unreliability with reference to acidity.—Medical Record, March 30, 1907.

